# Predicting University Students’ Stress Responses: The Role of Academic Stressors and Sociodemographic Variables

**DOI:** 10.3390/ejihpe15080163

**Published:** 2025-08-16

**Authors:** Cristina Ruiz-Camacho, Margarita Gozalo

**Affiliations:** 1 Department of Psychology and Anthropology, Faculty of Education and Psychology, University of Extremadura, 06071 Badajoz, Spain; 2 Department of Psychology and Anthropology, Faculty of Sport Science (Psychology Laboratory), University of Extremadura, 10005 Caceres, Spain

**Keywords:** stress responses, academic stressors, sociodemographic variables, university students, higher education, predictive analysis

## Abstract

**Background/Objectives:** Academic stress arises when students perceive that university demands exceed their coping resources, leading to cognitive, behavioral, and physiological stress responses. This study examines the predictive role of academic stressors and sociodemographic variables across five dimensions of stress response. **Methods:** The sample comprised 1014 Spanish university students (64.5% women, 35.5% men; *M* = 20.56, *SD* = 3.50). Participants completed the Academic Stressors Scale (E-CEA) and the Stress Responses Scale (R-CEA). Hierarchical regression analyses were conducted in two blocks: sociodemographic variables were entered in the first block, followed by academic stressors in the second. **Results:** Academic stressors accounted for substantial variance in all five stress response dimensions: negative thoughts (47.8%), physical exhaustion (39.5%), physical agitation (32.9%), irritability (29.7%), and sleep disturbances (26.8%). The most recurrent predictors were beliefs about performance, exams, and academic overload. In contrast, sociodemographic variables explained a much smaller portion of the variance (5.9%) and were specifically linked to higher irritability among women and younger students, more negative thoughts among students in Arts and Humanities, and fewer physical symptoms and sleep disturbances in students from science and technical fields. **Conclusions:** The findings reveal that academic stressors are key contributors to psychological distress among university students, highlighting the need for institutional interventions to alleviate the most frequent stressors. Identifying student groups particularly vulnerable to academic stress further supports the implementation of tailored strategies that address the diversity of students’ profiles and needs.

## 1. Introduction

University life constitutes a complex formative stage, characterized by significant changes in students’ academic, personal, and social environments. It involves not only access to new knowledge but also the need to assume responsibilities that demand autonomy, discipline, and continuous adaptation to increasingly demanding educational dynamics (Abarca et al., 2022; Noonan et al., 2024; Restrepo et al., 2023). This initial stage does not unfold uniformly: many students enter university without having fully developed the study strategies, self-regulation skills, and time management abilities required at this educational level, which may hinder their ability to cope effectively and increase their vulnerability to various sources of distress (Duche et al., 2020; Li et al., 2022; Reis et al., 2021). Epidemiological data estimate that between 47% and 55% of university students experience moderate levels of academic stress (Asensio-Martínez et al., 2023).

Although the transition to university has traditionally been identified as a critical moment (García-Ros et al., 2012; Román & Hernández, 2011), it is equally important to consider the ongoing adaptation processes that extend throughout the entire academic journey (Schlossberg, 2011). University represents a highly demanding environment, where personal, social, and structural factors converge, generating a constant burden of expectations that students must assess and manage (Asif et al., 2020). The recent literature consistently highlights that the accumulation of stressors within the university context can directly affect students’ psychological well-being, as well as their academic performance and persistence in higher education (Deng et al., 2022; Joseph et al., 2021; Liu et al., 2019).

### 1.1. Academic Stressors

Numerous studies have documented a wide range of stress-inducing factors in the university experience (Estrada-Araoz et al., 2024; Slimmen et al., 2022; Tito-Huamani et al., 2022), which can be classified, following the proposal of Muñoz (2004), into three major categories: (1) stressors related to assessment processes; (2) stressors linked to academic workload; and (3) stressors arising from the conditions of the teaching-learning process.

Among the first group, the most widely documented stressors in the literature are evaluation situations—particularly exams and oral presentations—which have been identified as critical triggers of anxiety, worry, and insecurity due to their direct influence on academic performance and future educational and professional opportunities (Al-Shahrani et al., 2023; Cabanach et al., 2016; Weber et al., 2019).

Regarding the second category, academic overload—whether due to the accumulation of tasks, deadline pressure, or the clustering of assessments within specific periods—is consistently associated with higher levels of stress, exhaustion, and demotivation (Chust-Hernández et al., 2022; Souto-Gestal et al., 2019). Alongside these stressors, beliefs about one’s academic performance represent another relevant factor: the self-perception of inadequacy or failure, especially when not moderated by constructive feedback, may give rise to dysfunctional thoughts that intensify distress in response to academic demands (Cabanach et al., 2016; Kristensen et al., 2023).

Finally, other stressors are related to the teaching-learning process, whose impact may be more moderate but enduring. Among these are teachers’ methodological deficiencies —such as lack of clarity in explanations, limited variety in instructional strategies, or the absence of clearly defined assessment criteria (Muñoz, 2004). Additional stressors include difficulties in active classroom participation, perceptions of a negative social climate, and students’ perceived lack of value regarding course content. Although these factors do not always produce high levels of stress, their persistence can gradually undermine students’ motivation and academic engagement (Ai et al., 2025; Johansen et al., 2023; Martincová & Bílá, 2023; Xethakis et al., 2024).

### 1.2. Theoretical Models

The transactional model of stress proposed by Lazarus and Folkman (1984) offers a robust theoretical framework for understanding how academic demands can become sources of distress. According to this model, stress does not lie in the situation itself but in the dynamic interaction between the individual and their environment, mediated by cognitive and emotional processes that shape how individuals cope. A perception of threat or overload arises when environmental demands are appraised as exceeding one’s personal or contextual resources.

In a complementary vein, the systemic–cognitive model developed by Barraza (2006, 2018) highlights that the experience of stress entails a disruption of equilibrium between the student and their educational environment. This dynamic and functional framework posits that academic stress emerges when students perceive themselves as lacking the resources necessary to cope with university demands. Such a negative appraisal leads to systemic imbalance, which in turn manifests as psychological distress and compromises overall student functioning.

### 1.3. Tripartite Response System

Building on the previous framework, Lang’s (1968) tripartite response system categorizes stress manifestations into three interrelated levels—physiological, psychological, and behavioral—offering a comprehensive understanding of how academic stress affects university students.

Physiological responses typically involve the activation of the sympathetic nervous system, resulting in symptoms such as excessive sweating, headaches, tachycardia, muscle tension, or increased respiratory rate. With prolonged exposure to stressors, sleep disturbances, digestive issues, and chronic fatigue may also emerge (Alqarni et al., 2025; Balmus et al., 2019; Fischer et al., 2016; McEwen & Akil, 2020).

Psychological symptoms include irritability, sadness, anxiety, demotivation, and cognitive difficulties, such as impaired concentration and memory. Under intense academic pressure, students often experience ruminative thoughts about performance and feelings of inefficacy (Cruz-Carabajal et al., 2024; García-Ros et al., 2012; Huang et al., 2020; Morrison & O’Connor, 2005; Shankar & Park, 2016).

Behavioral manifestations range from social withdrawal and absenteeism to procrastination and disruptions in sleep and eating patterns. In some cases, students resort to substances like tobacco, alcohol, or caffeine to cope with academic demands, particularly during exam periods (Belay-Ababu et al., 2018; Bezie et al., 2025; Mofatteh, 2021; Noonan et al., 2024; Restrepo et al., 2018; Zambrano et al., 2021).

### 1.4. Sociodemographic Variables in Academic Stress

The impact of stress responses among university students is not uniform; rather, it can be influenced by various factors that, although not direct triggers, affect the intensity, duration, or expression of stress (Crespo & Labrador, 2003). Among the most extensively studied are gender, age, academic year, and field of study—considered personal and educational variables that may shape differential vulnerability profiles to academic stress (Muñoz, 2004).

Gender differences have received particular attention. Numerous studies have indicated that women tend to report higher levels of academic stress than men, particularly in the emotional and cognitive dimensions of the stress experience (Gao et al., 2020; Graves et al., 2021; Karaman et al., 2019; Oliveira et al., 2020; Vidal-Conti et al., 2018). This disparity may be explained by coping styles that are less focused on actively managing academic demands and more oriented toward emotional regulation, as well as by higher self-imposed expectations or differential exposure to gender stereotypes within the educational environment (Cabanach et al., 2018; Matud et al., 2020).

Age has also been identified as a significant factor. Younger students—particularly those in the early years of their studies—tend to experience higher levels of emotional stress, irritability, and adjustment difficulties (Cassaretto et al., 2021; Córdova & Santa María, 2018; Restrepo et al., 2020). This increased vulnerability may be explained by the developmental transition involved in entering university, as well as by limited experience in managing complex academic demands (Arnett, 2000).

Similarly, academic year appears to play an influential role. First-year students often report greater uncertainty and lower perceived control, whereas academic progression tends to foster adaptation, the use of coping strategies, and reduced emotional reactivity (García-Ros et al., 2012). However, each stage of university involves specific stressors; in later years, academic overload and evaluative pressure are more prominent, along with potential dissatisfaction arising from a mismatch between initial expectations and academic reality (Abarca et al., 2022; Sabih et al., 2013).

Finally, field of study has emerged as another relevant dimension. Health-related programs show the highest stress levels, likely due to the combined demands of theoretical coursework and clinical practice (Labrague, 2024; Souto-Gestal et al., 2019). Nonetheless, substantial stress has also been reported among students in the Arts, Humanities, and Social Sciences (Casuso-Holgado et al., 2019; Noonan et al., 2024), as well as in Science and Technical fields with a strong mathematical component, which often present the highest dropout rates (Feser et al., 2023; Lahme et al., 2024).

### 1.5. The Present Study

Despite the substantial body of evidence regarding the influence of academic stressors on university students’ mental health, most studies have approached these factors in broad terms or by focusing on a single dimension, without analyzing the specific contribution of each stressor to the various types of stress responses—cognitive, physiological, or behavioral. This perspective limits the ability to accurately determine which academic situations are most strongly associated with the emergence of psychological distress.

In addition, few studies have simultaneously incorporated student characteristics—such as gender, age, or academic year—into integrated analytical models. While the predictive role of academic stressors, treated as a global construct, in academic stress has been acknowledged (Casuso-Holgado et al., 2019; Rogers et al., 2012), models capable of estimating the specific weight of individual stressors and sociodemographic variables in explaining students’ stress responses have yet to be developed.

Accordingly, the joint analysis of academic stressors and sociodemographic characteristics addresses the need to construct a more refined explanatory model. Whereas the former help identify which elements of the academic system function as sources of pressure, the latter offer key insights into who is most affected and under what conditions these pressures tend to exert a greater impact.

Within this framework, the present study aims to contribute to a more nuanced understanding of academic stress. Specifically, it examines the predictive capacity of various academic stressors—(a) teachers’ methodological deficiencies, (b) academic overload, (c) beliefs about performance, (d) public speaking situations, (e) unfavorable classroom climate, (f) perceived content irrelevance, (g) exams, and (h) participation difficulties—alongside socio-demographic variables (gender, age, academic year, and field of study), in relation to five types of stress responses: (a) negative thoughts, (b) irritability, (c) sleep disturbances, (d) physical exhaustion, and (e) physical agitation.

## 2. Methodology

### 2.1. Design and Participants

A descriptive, non-experimental study was conducted using a simple cross-sectional ex post facto design (Ato et al., 2013). The sample consisted of 1014 undergraduate students from the University of Extremadura, aged between 17 and 63 years (*M* = 20.56, *SD* = 3.50). A non-probability cluster sampling method was used, preserving the integrity of natural classroom groupings.

The inclusion criteria were being enrolled in an undergraduate program at the University of Extremadura during the 2024/2025 academic year and voluntary participation with informed consent. Although the study was primarily aimed at individuals over the age of 18, the participation of first-year students below this threshold was accepted, provided they met the above criteria and their participation adhered to the ethical principles of research.

Regarding gender, 64.5% of participants identified as female and 35.5% as male. The distribution across academic years was relatively balanced, with the largest proportion in the second year (28.80%), followed by the first (27.91%), fourth (23.27%), and third (20.02%). As for academic field, most students were enrolled in Social and Legal Sciences (43.0%) and Health Sciences (22.58%), with smaller percentages in Sciences (17.16%), Engineering and Architecture (8.78%), and Arts and Humanities (8.48%) (Table 1).

### 2.2. Measures

Academic stress was measured using two scales from the Academic Stress Questionnaire (CEA; Cabanach et al., 2008b):

- The Academic Stressors Scale (E-CEA, Cabanach et al., 2016) consists of 54 items assessing situations within the academic environment that may trigger stress among students. Responses are rated on a 5-point Likert scale ranging from 1 (never) to 5 (always). The scale comprises eight dimensions: (a) teachers’ methodological deficiencies (e.g., “I get nervous when the instructor does not clearly explain what we have to do”); (b) academic overload (e.g., “I get nervous about not having enough time to study all my subjects properly”); (c) beliefs about performance (e.g., “I get nervous because I do not think I can meet the demands of my degree program”); (d) public speaking situations (e.g., “I get nervous when I have to give a presentation or speak in front of others”); (e) unfavorable classroom climate (e.g., “I get nervous about the lack of support from classmates”); (f) perceived content irrelevance (e.g., “I get nervous when the subjects we study have little to do with my expectations”); (g) exams (e.g., “I get nervous as exam dates approach”); and (h) participation difficulties (e.g., “I get nervous because I am not allowed to decide how to approach my assignments or tasks”). Previous studies have reported high internal consistency for this scale, with Cronbach’s alpha values ranging from 0.83 to 0.96 across dimensions (Casuso-Holgado et al., 2019; Souto-Gestal et al., 2019).
- The Stress Response Scale (R-CEA; Cabanach et al., 2008a) assesses the symptoms associated with academic stress, encompassing physiological, cognitive, and behavioral manifestations. This scale includes 22 items rated on a 5-point Likert scale from 1 (never) to 5 (always), indicating the frequency with which students experience these symptoms. The R-CEA is structured into five dimensions: (a) negative thoughts (e.g., “In recent weeks, I have tended to focus on my failures and minimize my successes”); (b) irritability (e.g., “In recent weeks, I have felt irritated by even small inconveniences”); (c) sleep disturbances (e.g., “In recent weeks, I have had trouble falling asleep”); (d) physical exhaustion (e.g., “In recent weeks, I have felt easily fatigued”); and (e) physical agitation (e.g., “In recent weeks, I have experienced heart palpitations”). The R-CEA has shown adequate reliability in previous research, with Cronbach’s alpha coefficients ranging from 0.85 to 0.94 across dimensions (Cabanach et al., 2018; Casuso-Holgado et al., 2019).

Sociodemographic variables were assessed in a brief online self-report section. Gender was coded as a dichotomous variable (0 = male; 1 = female). Age and year of study were entered as continuous variables (in years and ranging from first to fourth year, respectively). Academic field was treated as a categorical predictor using dummy coding, with Social and Legal Sciences as the reference category; the comparison groups were Health Sciences, Arts and Humanities, Sciences, and Engineering and Architecture.

### 2.3. Procedure

Data were collected between February and April 2025 via an online survey administered through Google Forms, which included different sections containing the study scales and sociodemographic questions. Participants accessed the questionnaire by scanning a QR code distributed within their respective faculties. Anonymity and confidentiality were ensured throughout the entire process. Before participating, students provided informed consent after receiving information about the study’s objectives and the voluntary nature of their involvement. To minimize social desirability bias, participants were reminded that there were no right or wrong answers. Completing the questionnaire took approximately 20 min. This study was conducted in accordance with the ethical principles outlined in the Declaration of Helsinki and the Ethical Code of the University of Extremadura.

### 2.4. Data Analysis

Cronbach’s alpha coefficients were initially computed to evaluate the internal consistency of the dimensions related to academic stressors and stress responses. Subsequently, the normality of the data was assessed: univariate normality was examined using the Kolmogorov–Smirnov test, while multivariate normality was evaluated through Mardia’s test.

Descriptive statistics were then calculated for each study dimension. To explore bivariate associations between academic stressors and stress responses, Pearson correlation coefficients were computed.

Finally, hierarchical regression analyses were conducted in two steps for each stress response dimension. In the first step, sociodemographic variables were included to estimate their explanatory power. In the second step, academic stressors were added to assess their incremental contribution to the prediction.

All analyses were performed using SPSS software (version 26.0), with the significance level set at *p* < 0.05.

## 3. Results

### 3.1. Initial Analyses

As shown in Table 2, all dimensions of academic stressors and stress responses exhibited good internal consistency, with Cronbach’s alpha coefficients ranging from 0.79 to 0.88. Among the stressor dimensions, the highest mean scores were found for exams, academic overload, and teachers’ methodological deficiencies. For stress responses, physical exhaustion and negative thoughts recorded the highest means.

Significant and positive correlations were observed across most dimensions. Particularly strong associations emerged between negative thoughts and beliefs about performance (*r* = 0.63, *p* < 0.01), physical exhaustion, and academic overload (*r* = 0.55, *p* < 0.01) and negative thoughts and academic overload (*r* = 0.47, *p* < 0.01).

### 3.2. Hierarchical Multiple Regression Analysis

Table 3 details the results for the negative thoughts dimension. In Step 1, identifying as female (*β* = 0.14, *p* < 0.001) and studying Health Sciences (*β* = 0.12, *p* < 0.001), Arts and Humanities (*β* = 0.16, *p* < 0.001), or Sciences (*β* = 0.19, *p* < 0.001) were significant predictors of negative thoughts. With the inclusion of academic stressors in Step 2, the proportion of explained variance increased to 47.8% (Δ*R*^2^ = 42.6%). At this point, Arts and Humanities remained a significant predictor (*β* = 0.09, *p* < 0.001), indicating an increased vulnerability to negative thoughts compared to the reference group. The strongest stressor predictors were beliefs about performance (*β* = 0.44, *p* < 0.001), public speaking situations (*β* = 0.14, *p* < 0.001), and exams (*β* = 0.08, *p* = 0.008). Academic overload (*β* = 0.05, *p* = 0.139) and perceived content irrelevance (*β* = 0.05, *p* = 0.094) did not emerge as significant predictors.

Table 4 reports the regression outcomes for the irritability dimension. In Step 1, identifying as female (*β* = 0.19, *p* < 0.001), being in higher academic years (*β* = 0.08, *p* = 0.018), and pursuing degrees in Health Sciences (*β* = 0.14, *p* < 0.001) or Arts and Humanities (*β* = 0.09, *p* = 0.007) were significant predictors of irritability. After incorporating academic stressors in Step 2, the explained variance rose to 29.7% (Δ*R*^2^ = 23.8%). Gender (*β* = 0.08, *p* = 0.013) and age (*β* = −0.07, *p* = 0.018) remained significant, indicating greater irritability among younger students and women. The most influential academic stressors were unfavorable classroom climate (*β* = 0.15, *p* < 0.001), beliefs about performance (*β* = 0.14, *p* < 0.001), and academic overload (*β* = 0.11, *p* = 0.003). In contrast, perceived content irrelevance (*β* = 0.04, *p* = 0.288) and participation difficulties (*β* = 0.03, *p* = 0.348) did not show significant effects.

Table 5 presents the results for the sleep disturbances dimension. In Step 1, identifying as female (*β* = 0.09, *p* = 0.005), being in higher academic years (*β* = 0.09, *p* = 0.006), and studying Health Sciences (*β* = 0.07, *p* = 0.002) or Arts and Humanities (*β* = 0.11, *p* = 0.001) emerged as significant predictors of sleep difficulties. With the inclusion of academic stressors in Step 2, the explained variance increased to 26.8% (Δ*R*^2^ = 24.4%). At this point, studying Sciences was a significant negative predictor (*β* = −0.09, *p* = 0.002), suggesting fewer sleep difficulties compared to the reference group. The strongest stressor predictors were beliefs about performance (*β* = 0.15, *p* < 0.001), exams (*β* = 0.13, *p* < 0.001), and academic overload (*β* = 0.12, *p* = 0.002), while teachers’ methodological deficiencies (*β* = 0.01, *p* = 0.720) and public speaking (*β* = 0.06, *p* = 0.059) were not significant.

Table 6 summarizes the results of the hierarchical regression model for the physical exhaustion dimension. In Step 1, identifying as female (*β* = 0.16, *p* < 0.001) and studying Health Sciences (*β* = 0.11, *p* = 0.002) emerged as significant predictors of physical exhaustion. Following the inclusion of academic stressors in Step 2, the model accounted for 39.5% of the variance (Δ*R*^2^ = 36.8%). At this stage, studying Sciences appeared as a significant negative predictor (*β* = −0.07, *p* = 0.014), suggesting a lower tendency toward physical exhaustion compared to the reference group (Social and Legal Sciences). Among the academic stressors, the strongest predictors were academic overload (*β* = 0.27, *p* < 0.001), beliefs about performance (*β* = 0.15, *p* < 0.001), and public speaking situations (*β* = 0.15, *p* < 0.001). In contrast, unfavorable classroom climate (*β* = 0.00, *p* = 0.998) and perceived irrelevance of academic content (*β* = 0.04, *p* = 0.195) did not show significant effects.

Table 7 displays the findings for the physical agitation dimension. In Step 1, both gender and academic field were significant predictors: identifying as female (*β* = 0.13, *p* < 0.001) and being enrolled in Health Sciences (*β* = 0.10, *p* = 0.003), Arts and Humanities (*β* = 0.10, *p* = 0.002), or Sciences (*β* = 0.10, *p* = 0.004) were significantly associated with greater physical agitation. After including academic stressors in Step 2, the explained variance increased to 32.9% (Δ*R*^2^ = 29.1%), with Engineering and Architecture emerging as a significant negative predictor (*β* = −0.06, *p* = 0.031), suggesting lower physical agitation in this field compared to the reference group. Among the academic stressors, the most influential were exams (*β* = 0.17, *p* < 0.001), beliefs about performance (*β* = 0.15, *p* < 0.001), and academic overload (*β* = 0.12, *p* = 0.002).

## 4. Discussion

The aim of the present study was to analyze the contribution of various academic stressors and the role of sociodemographic variables in predicting stress responses among university students. The results obtained enhance our understanding of the factors influencing the cognitive, behavioral, and physiological manifestations of academic stress, highlighting the specific contribution of each.

Hierarchical regression analyses confirmed that academic stressors are the primary predictors of stress responses. Their progressive inclusion in the models significantly increased the explained variance across all assessed dimensions, while substantially reducing the explanatory power attributed to sociodemographic variables. The following section discusses the specific results for each dimension of stress responses evaluated.

Negative thoughts represented the dimension with the highest percentage of variance explained by the set of academic stressors (*R*^2^ = 42.6%), with beliefs about academic performance emerging as the strongest individual predictor. This finding suggests a particularly close link between cognitive factors and this manifestation of academic distress, consistent with previous studies highlighting the role of self-referential cognitions—such as doubts about one’s own competence or fear of failure—in the experience of stress within the university context (Inostroza et al., 2024; Navarro-Mateu et al., 2020). Additionally, stressors related to public speaking and exams showed substantial explanatory power in this dimension, possibly indicating that frequent exposure to evaluative situations, both oral and written, is associated with increased activation of anticipatory or intrusive thoughts (Kollárik et al., 2022).

Regarding sociodemographic variables within this dimension, the initial model (*R*^2^ = 5.2%) indicated that being female and pursuing studies in Health Sciences, Arts and Humanities, or Sciences was associated with a greater presence of negative thoughts. However, after the inclusion of academic stressors, most of these effects lost statistical significance, with only the Arts and Humanities area remaining a relevant predictor. This finding suggests that the differences observed in the first step of the model may be partly explained by differential exposure to stress-inducing situations within the academic context. Nevertheless, the residual effect observed among students in Arts and Humanities may reflect specific characteristics of this field—such as professional uncertainty or the evaluative nature of creative work—that could intensify the emergence of negative thoughts (Oh & Kim, 2020; Quadlin, 2017).

For the irritability dimension, academic stressors explained 23.8% of the variance, with beliefs about academic performance and a negative social climate emerging as the most influential predictors. This association suggests that both self-imposed expectations and the perception of an academic environment marked by competitiveness or tense interpersonal dynamics could be related to greater difficulties in students’ emotional regulation (Chambel & Curral, 2005; Fierro-Suero et al., 2021). Likewise, academic overload showed a significant contribution to this response, which might reflect that an accumulation of academic demands is associated with increased affective reactivity in the university context (Padrón et al., 2021).

Regarding the role of sociodemographic variables in this dimension, the initial model (*R*^2^ = 5.9%) showed that being female, studying in advanced academic years, and belonging to the Health Sciences or Arts and Humanities fields was associated with higher levels of irritability. However, after the inclusion of academic stressors, these effects were attenuated, losing statistical significance in all cases except for gender. In addition, age emerged as a negative predictor, suggesting greater emotional reactivity among younger students (Arnett, 2000; Dungog et al., 2021; Murphy et al., 2019). This pattern may indicate that the initial differences are partly explained by unequal exposure to specific stressors. The persistence of the gender effect, however, points to the possibility that other differential factors—biological, social, or related to emotional socialization—may influence the tendency toward irritability in academic settings (Bezie et al., 2025; Chust-Hernández et al., 2022; Ramón-Arbués et al., 2020; Reddy et al., 2018).

Sleep disturbances were also notably explained by academic stressors, accounting for 24.4% of the variance. Among them, beliefs about academic performance, exams, and academic overload emerged as the most relevant predictors. These results suggest that exposure to cognitively demanding tasks and evaluative situations could be linked to a higher likelihood of experiencing difficulties in achieving restorative sleep. This interpretation aligns with previous studies that associate such stressors with increased mental and emotional activation before nighttime rest (Dagani et al., 2024; Schmickler et al., 2023).

As for the role of sociodemographic variables in this dimension, the initial model (R^2^ = 2.4%) indicated a greater presence of sleep disturbances among women, students in advanced academic years, and those enrolled in Health Sciences or Arts and Humanities programs. However, after academic stressors were included in the model, most of these effects lost statistical significance, with only a lower propensity observed among students in the Sciences field remaining significant. While this finding is suggestive, the available literature remains limited and heterogeneous, warranting cautious interpretation (Kuhn et al., 2024; Schmickler et al., 2023).

Physical manifestations of stress were also significantly explained by academic stressors, accounting for 36.8% of the variance in physical exhaustion and 29.1% in physical agitation. In both types of responses, exams and academic overload emerged as the most influential stressors. This association suggests that increased exposure to evaluative demands and excessive workloads may be linked to higher levels of somatic symptoms among university students (Fischer et al., 2016; Williams-York et al., 2024).

When considering sociodemographic variables, a pattern similar to that observed in previous dimensions emerged, with gender and field of study exerting a greater initial impact: women and students in Health Sciences or Arts and Humanities tended to report more physical symptoms. However, after the inclusion of academic stressors, most of these effects lost statistical significance. At this stage, students enrolled in Science or Engineering and Architecture programs showed a lower propensity to experience physical manifestations of distress. This difference could be related to contextual characteristics of these fields, such as a more favorable perception of future employability or lower vocational uncertainty (Ferrer-Pérez et al., 2023; Stiglic et al., 2022). Nonetheless, due to the scarcity of specific studies in this area, these interpretations should be viewed with caution.

### 4.1. Theoretical and Practical Implications

From a theoretical perspective, our findings reinforce the understanding of academic stress as a multidimensional phenomenon shaped by the interplay of individual and contextual factors. Although academic stressors accounted for the largest share of variance across all stress responses, sociodemographic variables also contributed explanatory value in certain cases, supporting the need for transactional and systemic approaches to stress (Barraza, 2018; Lazarus & Folkman, 1984).

However, it is crucial to distinguish the distal, structural nature of sociodemographic variables—whose influence tends to be indirect or mediated—from the proximal, situational nature of academic stressors, which are more amenable to educational intervention. Treating both sets of factors as if they operated at the same explanatory level risks a reductionist reading of the sources of student distress.

Building on this conceptual framework, sociodemographic variables require broader institutional policies—such as the implementation of financial aid schemes, academic support programs, and guidance services—to address structural sources of vulnerability (Gibson-Smith et al., 2025). Alongside these broader measures, our findings suggest that women, younger students, and those enrolled in Arts-related programs may be particularly susceptible to emotional distress, underscoring the relevance of targeted interventions. For female students, gender-sensitive mentoring networks and workshops focused on self-efficacy and emotional regulation may enhance their ability to manage academic demands more effectively. For younger students, early-stage transition programs incorporating time management, study strategies, and brief mindfulness techniques could help reduce irritability and foster more adaptive coping. Finally, for Arts students, small-group interventions centered on cognitive reframing, emotional expression, and academic self-concept may support the reduction in maladaptive thinking patterns and the development of psychological resilience within creative learning environments.

In parallel, academic stressors are more amenable to direct educational intervention and can be addressed through targeted curricular and pedagogical strategies. The most relevant approaches include the following (Chambel & Curral, 2005; Muñoz, 2004; Vizoso & Arias, 2016):

- Enhancing curricular planning by distributing assessment deadlines and examinations more evenly across the semester, thereby helping to reduce academic overload, fatigue, and emotional strain.
- Implementing active learning methodologies that promote student autonomy and decision-making, strengthen perceived control, and mitigate negative thoughts related to academic performance.
- Revising assessment systems to incorporate continuous and formative approaches that diversify evaluation formats and moderate the weight of high-stakes exams and oral presentations, with the aim of reducing evaluation-related anxiety and fostering more sustained learning.
- Fostering a positive academic climate through peer mentoring, constructive feedback, and inclusive integration activities that reinforce students’ sense of belonging and overall well-being.

These proposals are particularly relevant considering that, in our study, the most recurrent stressors were linked to beliefs about academic performance, academic overload, and exams—areas that are directly related to pedagogical practices and, therefore, amenable to improvement through educational intervention.

### 4.2. Limitations and Future Research Directions

Although this study adopts an innovative and thorough approach to academic stress from a dimensional perspective—allowing for the analysis of how specific stressors and individual characteristics predict distinct manifestations of distress—it presents several limitations. First, the cross-sectional design prevents the establishment of causal relationships and limits the examination of how stress responses evolve over time. Second, the exclusive use of self-report measures may have introduced social desirability bias. Third, data were collected from a single university, which restricts the generalizability of the findings to other educational contexts. Fourth, other relevant contextual variables (e.g., socioeconomic status, type of residence, or external workload) were not included, despite their potential contribution to explaining differences in stress responses. Lastly, although sociodemographic variables were considered as predictors, their potential moderating role was not explored.

Future research should consider employing longitudinal designs to better capture the temporal dynamics of academic stress and investigate potential causal pathways. It is also recommended to incorporate additional data collection techniques, such as in-depth interviews, and to broaden the sample to include universities from more diverse sociocultural settings. Advanced analytical models, such as structural equation modeling, could be used to further examine the relationships identified in this study and to incorporate psychosocial variables—such as self-efficacy or social support—that may function as protective factors against stress. Finally, intervention studies are needed to assess the impact of institutional programs aimed at both reducing stressors and enhancing students’ coping resources.

## 5. Conclusions

The results of the present study highlight the central role played by academic stressors in shaping university-related stress, particularly underscoring the high frequency and impact of beliefs about academic performance, academic overload, and examinations. These factors were significantly associated with various manifestations of psychological distress—including physical exhaustion, negative thoughts, sleep disturbances, irritability, and physical agitation—although their specific weight varied depending on the dimension analyzed.

This pattern of findings underscores the urgency of implementing institutional interventions aimed at mitigating the most prevalent stressors through improved curricular planning, the adoption of participatory teaching methodologies, the enhancement of the academic climate, and the strengthening of student support systems. Coordinated action across teaching and university management could substantially reduce levels of psychological distress and foster more equitable and sustainable learning environments.

In addition to academic factors, certain sociodemographic variables were also found to influence specific dimensions of stress, thereby contributing to a more nuanced understanding of the phenomenon. Although their overall influence was relatively limited, higher levels of distress were observed among women, younger students, and those enrolled in Arts and Humanities programs. Taking these differences into account may be key to designing preventive strategies tailored to the specific characteristics and needs of each group.

Taken together, the findings reinforce the need to adopt an integrative perspective in the study and management of academic stress—one that combines structural changes within the educational system with targeted actions adapted to the diversity of the university student population.

## Figures and Tables

**Table 1:** Sociodemographic and academic characteristics of the sample (*N* = 1014).

Variables	*n*	%
Gender		
Female	654	64.50
Male	360	35.50
Age		
Range	17–63	
Mean	20.56	
Study Year		
First	283	27.91
Second	292	28.80
Third	203	20.02
Fourth	236	23.27
Academic Field		
Social and Legal Sciences	436	43.00
Health Sciences	229	22.58
Arts and Humanities	86	8.48
Sciences	174	17.16
Engineering and Architecture	89	8.78

**Table 2:** Cronbach’s alpha, descriptive statistics, and bivariate correlations between the dimensions of the E-CEA and R-CEA scales.

Variables	α	*M* (*SD*)	1	2	3	4	5	6	7	8	9	10	11	12
1. TMD	0.84	3.08 (1.04)	1											
2. AO	0.88	3.11 (1.09)	0.45 *	1										
3. BAP	0.86	2.53 (1.06)	0.31 *	0.54 *	1									
4. PSS	0.84	2.90 (1.07)	0.17	0.27 *	0.33 *	1								
5. UCC	0.79	1.91 (0.76)	0.38 *	0.41 *	0.30 *	0.12	1							
6. PCI	0.79	2.26 (0.91)	0.45 *	0.37 *	0.39 *	0.08	0.44 *	1						
7. EXAM	0.84	3.45 (1.04)	0.47 *	0.56 *	0.47 *	0.21 *	0.32 *	0.29 *	1					
8. PA	0.85	2.38 (1.06)	0.47 *	0.48 *	0.31 *	0.07	0.55 *	0.47 *	0.39 *	1				
9. PE	0.82	2.94 (0.95)	0.41 *	0.55 *	0.46 *	0.32 *	0.31 *	0.32 *	0.44 *	0.38 *	1			
10. SD	0.82	2.37 (0.81)	0.31 *	0.40 *	0.38 *	0.18 *	0.36 *	0.35 *	0.37 *	0.37 *	0.61 *	1		
11. IRRIT	0.87	2.41 (0.89)	0.37 *	0.42 *	0.39 *	0.24 *	0.37 *	0.30 *	0.39 *	0.33 *	0.55 *	0.54 *	1	
12. NT	0.85	2.72 (1.02)	0.36 *	0.47 *	0.63 *	0.34 *	0.35 *	0.37 *	0.44 *	0.36 *	0.58 *	0.54 *	0.55 *	1
13. PA	0.81	2.39 (0.87)	0.38 *	0.45 *	0.42 *	0.22 *	0.37 *	0.35 *	0.45 *	0.39 *	0.61 *	0.67 *	0.61 *	0.59 *

Note. TMD = teachers’ methodological deficiencies; AO = academic overload; BAP = beliefs about performance; PSS = public speaking situations; UCC = unfavorable classroom climate; PCI = perceived content irrelevance; EXAM = exams; PA = participation difficulties; PE = physical exhaustion; SD = sleep disturbances; IRRIT = irritability; NT = negative thoughts; PA = physical agitation; α = Cronbach’s alpha; *M* = mean; *SD* = standard deviation; * *p* < 0.01.

**Table 3:** Regression analysis for negative thoughts.

Predictor	Step 1			Step 2		
	*B (SE)*	*β*	*t*	*B (SE)*	*β*	*t*
Gender (Female vs. Male)	0.29 (0.07)	0.14	4.20 ***	−0.05 (0.06)	−0.03	−0.97
Age	−0.02 (0.01)	−0.06	−1.77	−0.01 (0.01)	−0.04	−1.56
Study Year	0.03 (0.03)	0.03	0.97	−0.02 (0.02)	−0.02	−0.82
Academic Field						
Social and Legal Sciences	Ref.					
Health Sciences	0.29 (0.08)	0.12	3.51 ***	0.02 (0.06)	0.01	0.30
Arts and Humanities	0.58 (0.12)	0.16	4.90 ***	0.33 (0.09)	0.09	3.65 ***
Sciences	0.51 (0.09)	0.19	5.63 ***	0.12 (0.07)	0.05	1.75
Engineering and Architecture	0.13 (0.12)	0.04	1.07	−0.05 (0.09)	−0.02	−0.59
E-CEA						
Teachers’ methodological deficiencies				0.06 (0.03)	0.06	2.05 *
Academic overload				0.05 (0.03)	0.05	1.48
Beliefs about performance				0.42 (0.03)	0.44	14.60 ***
Public speaking situations				0.14 (0.02)	0.14	5.67 ***
Unfavorable classroom climate				0.09 (0.04)	0.07	2.43 *
Perceived content irrelevance				0.06 (0.03)	0.05	1.68
Exams				0.08 (0.03)	0.08	2.65 **
Participation difficulties				0.07 (0.03)	0.07	2.27 *
*R*^2^ (%)	5.2			47.8		
∆*R*^2^ (%)				42.6 ***		
Model	*F*(7,1013) = 8.94, *p* < 0.001			*F*(15,1013) = 62.87, *p* < 0.001		

Note. *B* = unstandardized regression coefficient; *SE* = standard error; *β* = standardized regression coefficient*;* * *p* < 0.05; ** *p* < 0.01; *** p < 0.001.

**Table 4:** Regression analysis for irritability.

Predictor	Step 1			Step 2		
	*B (SE)*	*β*	*t*	*B (SE)*	*β*	*t*
Gender (Female vs. Male)	0.36 (0.06)	0.19	5.93 ***	0.14 (0.06)	0.08	2.50 *
Age	−0.02 (0.01)	−0.06	−1.95	−0.02 (0.01)	−0.07	−2.38 *
Study Year	0.06 (0.03)	0.08	2.38 *	0.01 (0.02)	0.01	0.49
Academic Field						
Social and Legal Sciences	Ref.					
Health Sciences	0.30 (0.07)	0.14	4.23 ***	0.12 (0.06)	0.06	1.86
Arts and Humanities	0.28 (0.10)	0.09	2.70 **	0.08 (0.09)	0.03	0.92
Sciences	0.08 (0.08)	0.03	1.03	−0.12 (0.07)	−0.05	−1.69
Engineering and Architecture	−0.07 (0.11)	−0.02	−0.62	−0.16 (0.09)	−0.05	−1.72
E-CEA						
Teachers’ methodological deficiencies				0.09 (0.03)	0.10	3.28 **
Academic overload				0.09 (0.03)	0.11	2.96 **
Beliefs about performance				0.12 (0.03)	0.14	4.10 ***
Public speaking situations				0.06 (0.02)	0.07	2.38 *
Unfavorable classroom climate				0.17 (0.04)	0.15	4.40 ***
Perceived content irrelevance				0.04 (0.03)	0.04	1.06
Exams				0.09 (0.03)	0.10	3.03 **
Participation difficulties				0.03 (0.03)	0.03	0.94
*R*^2^ (%)	5.9			29.70		
∆*R*^2^ (%)				23.8 ***		
Model	*F*(7,1013) = 10.08, *p* < 0.001			*F*(15,1013) = 29.58, *p* < 0.001		

Note. *B* = unstandardized regression coefficient; *SE* = standard error; *β* = standardized regression coefficient*;* * *p* < 0.05; ** *p* < 0.01; *** p < 0.001.

**Table 5:** Regression analysis for sleep disturbances.

Predictor	Step 1			Step 2		
	*B (SE)*	*β*	*t*	*B (SE)*	*β*	*t*
Gender (Female vs. Male)	0.16 (0.06)	0.09	2.83 **	−0.02 (0.05)	−0.01	−0.34
Age	0.00 (0.01)	0.00	0.04	0.00 (0.01)	−0.01	−0.24
Study Year	0.07 (0.02)	0.09	2.77 **	0.02 (0.02)	0.03	0.93
Academic Field						
Social and Legal Sciences	Ref.					
Health Sciences	0.14 (0.07)	0.07	2.08 *	−0.02 (0.06)	−0.01	−0.38
Arts and Humanities	0.31 (0.10)	0.11	3.22 **	0.12 (0.09)	0.04	1.38
Sciences	−0.01 (0.07)	0.00	−0.08	−0.20 (0.07)	−0.09	−3.06 **
Engineering and Architecture	0.05 (0.10)	0.02	0.54	−0.04 (0.09)	−0.01	−0.46
E-CEA						
Teachers’ methodological deficiencies				0.01 (0.03)	0.01	0.36
Academic overload				0.09 (0.03)	0.12	3.18 **
Beliefs about performance				0.11 (0.03)	0.15	4.17 ***
Public speaking situations				0.04 (0.02)	0.06	1.92
Unfavorable classroom climate				0.11 (0.04)	0.11	3.04 **
Perceived content irrelevance				0.09 (0.03)	0.10	2.96 **
Exams				0.10 (0.03)	0.13	3.58 ***
Participation difficulties				0.07 (0.03)	0.08	2.29 *
*R*^2^ (%)	2.40			26.8		
∆*R*^2^ (%)				24.4 ***		
Model	*F*(7,1013) = 4.49; *p* < 0.001			*F*(15,1013) = 25.77; *p* < 0.001		

Note. *B* = unstandardized regression coefficient; *SE* = standard error; *β* = standardized regression coefficient*;* * *p* < 0.05; ** *p* < 0.01; *** p < 0.001.

**Table 6:** Regression analysis for physical exhaustion.

Predictor	Step 1			Step 2		
	*B (SE)*	*β*	*t*	*B (SE)*	*β*	*t*
Gender (Female vs. Male)	0.31 (0.07)	0.16	4.76 ***	−0.02 (0.06)	−0.01	−0.28
Age	−0.01 (0.01)	−0.05	−1.38	−0.01 (0.01)	−0.04	−1.38
Study Year	0.05 (0.03)	0.06	1.75	−0.01 (0.02)	−0.01	−0.43
Academic Field						
Social and Legal Sciences	Ref.					
Health Sciences	0.24 (0.08)	0.11	3.11 **	−0.03 (0.06)	−0.01	−0.52
Arts and Humanities	0.11 (0.11)	0.03	1.01	−0.07 (0.09)	−0.02	−0.72
Sciences	0.16 (0.09)	0.06	1.83	−0.17 (0.07)	−0.07	−2.46 *
Engineering and Architecture	0.08 (0.12)	0.02	0.69	−0.10 (0.09)	−0.03	−1.11
E-CEA						
Teachers’ methodological deficiencies				0.10 (0.03)	0.11	3.56 ***
Academic overload				0.24 (0.03)	0.27	7.68 ***
Beliefs about performance				0.14 (0.03)	0.15	4.72 ***
Public speaking situations				0.13 (0.02)	0.15	5.56 ***
Unfavorable classroom climate				0.00 (0.04)	0.00	0.00
Perceived content irrelevance				0.04 (0.03)	0.04	1.30
Exams				0.09 (0.03)	0.10	3.11 **
Participation difficulties				0.08 (0.03)	0.09	2.73 **
*R*^2^ (%)	2.7			39.5		
∆*R*^2^ (%)				36.8 ***		
Model	*F*(7,1013) = 5.03, *p* < 0.001			*F*(15,1013) = 45.03, *p* < 0.001		

Note. *B* = unstandardized regression coefficient; *SE* = standard error; *β* = standardized regression coefficient*;* * *p* < 0.05; ** *p* < 0.01; *** p < 0.001.

**Table 7:** Regression analysis for physical agitation.

Predictor	Step 1			Step 2		
	*B (SE)*	*β*	*t*	*B (SE)*	*β*	*t*
Gender (Female vs. Male)	0.24 (0.06)	0.13	4.07 ***	0.03 (0.05)	0.02	0.50
Age	0.00 (0.01)	0.02	0.54	0.00 (0.01)	0.01	0.43
Study Year	0.04 (0.03)	0.06	1.77	−0.01 (0.02)	−0.01	−0.22
Academic Field						
Social and Legal Sciences	Ref.					
Health Sciences	0.21 (0.07)	0.10	3.02 **	0.02 (0.06)	0.01	0.34
Arts and Humanities	0.32 (0.10)	0.10	3.12 **	0.10 (0.09)	0.03	1.10
Sciences	0.22 (0.08)	0.10	2.86 **	−0.01 (0.07)	0.00	−0.07
Engineering and Architecture	−0.09 (0.11)	−0.03	−0.81	−0.19 (0.09)	−0.06	−2.16 *
E-CEA						
Teachers’ methodological deficiencies				0.06 (0.03)	0.07	2.05 *
Academic overload				0.09 (0.03)	0.12	3.12 **
Beliefs about performance				0.12 (0.03)	0.15	4.44 ***
Public speaking situations				0.05 (0.02)	0.06	2.10 *
Unfavorable classroom climate				0.11 (0.04)	0.09	2.80 **
Perceived content irrelevance				0.07 (0.03)	0.07	2.19 *
Exams				0.14 (0.03)	0.17	5.16 ***
Participation difficulties				0.08 (0.03)	0.09	2.62 **
*R*^2^ (%)	3.8			32.9		
∆*R*^2^ (%)				29.1 ***		
Model	*F*(7,1013) = 6.70, *p* < 0.001			*F*(15,1013) = 34.18, *p* < 0.001		

Note. *B* = unstandardized regression coefficient; *SE* = standard error; *β* = standardized regression coefficient*;* * *p* < 0.05; ** *p* < 0.01; *** p < 0.001.

## Data Availability

The data are available upon request to the corresponding author.
